# Goldmann applanation tonometry error relative to true intracameral intraocular pressure in vitro and in vivo

**DOI:** 10.1186/s12886-017-0608-y

**Published:** 2017-11-25

**Authors:** Sean McCafferty, Jason Levine, Jim Schwiegerling, Eniko T. Enikov

**Affiliations:** 1 Intuor Technolgies, LLC, 6422 E. Speedway Blvd. Tucson, Suite 100, Tucson, AZ 85710 USA; 2 Department of Ophthalmology, University of Arizona College of Medicine, 655 n alvernon, Tucson, AZ 85710 USA; 3 Department of Mechanical and Aerospace, University of Arizona College of Engineering, 1130 N. Mountain Ave., Tucson, AZ 85721 USA; 4 University of Arizona College of Optical Science, 1630 E. University Blvd., Tucson, AZ 85719 USA; 5 Arizona Eye Consultants, 6422 E. Speedway Blvd. Tucson, Suite 100, Tucson, AZ 85710 USA

**Keywords:** Glaucoma, Intraocular pressure, IOP, Goldmann, Bias, Error, Perkins, Tonometer, Applanation, CCT, Central corneal thickness, CRF, Corneal resistance factor, Intracameral, Cadaver eye, In vivo, In vitro, Head position, Upright, Supine, Manometric, Corneal hydration

## Abstract

**Background:**

Goldmann applanation tonometry (GAT) error relative to intracameral intraocular pressure (IOP) has not been examined comparatively in both human cadaver eyes and in live human eyes. Futhermore, correlations to biomechanical corneal properties and positional changes have not been examined directly to intracameral IOP and GAT IOP.

**Methods:**

Intracameral IOP was measured via pressure transducer on fifty-eight (58) eyes undergoing cataract surgery and the IOP was modulated manometrically on each patient alternately to 10, 20, and 40 mmHg. IOP was measured using a Perkins tonometer in the supine position on 58 eyes and upright on a subset of 8 eyes. Twenty one (21) fresh human cadaver globes were Intracamerally IOP adjusted and measured via pressure transducer. Intracameral IOP ranged between 5 and 60 mmHg. IOP was measured in the upright position with a Goldmann Applanation Tonometer (GAT) and supine position with a Perkins tonometer. Central corneal thickness (CCT) was also measured.

**Results:**

The Goldmann-type tonometer error measured on live human eyes was 5.2 +/−1.6 mmHg lower than intracameral IOP in the upright position and 7.9 +/− 2.3 mmHg lower in the supine position (*p* < .05). CCT also indicated a sloped correlation to error (correlation coeff. = 0.18). Cadaver eye IOP measurements were 3.1+/−2.5 mmHg lower than intracameral IOP in the upright position and 5.4+/− 3.1 mmHg in the supine position (*p* < .05).

**Conclusion:**

Goldmann IOP measures significantly lower than true intracameral IOP by approximately 3 mmHg in vitro and 5 mmHg in vivo. The Goldmann IOP error is increased an additional 2.8 mmHg lower in the supine position. CCT appears to significantly affect the error by up to 4 mmHg over the sample size.

## Translational relevance

Verifies and quantifies IOP errors seen in previous theoretical modeling.

## What was known

1. Overall bias and biomechanical errors in Goldmann tonometry exist. Debate exists about how much.
2. Patient positional errors exist, but again difficult to quantify.
3. Unknown comparison quality in studies using cadaver and live human eyes.

## What this paper demonstrates

1. Quantifies statistically the overall error in Goldmann tonometry to true intracameral IOP
2. Live human eye manometric adjustment and maintenance of intracameral IOP at three (3) separate physiological values compared simultaneously to applanation tonometer IOP measurements.
3. Good Correlation in results is demonstrated between fresh cadaver eyes and live human eyes.
4. Quantifies statistically upright to supine positional error to a modulated intracameral IOP.
5. Demonstrates effect of CCT in live human eyes and corneal hydration in cadaver eyes on Goldmann IOP error.

## Background

Intraocular pressure (IOP) is a key objective measure in the diagnosis and treatment of glaucoma [1, 2]. Also, there are many other conditions in which IOP can be clinically relavent [1–3]. Goldmann Applanation Tonometry (GAT) remains the gold standard for IOP measurement [4]. Errors in Goldmann IOP measurements have been identified due to variability in corneal biomechanics [5–8]. Central corneal thickness (CCT) correction is an incomplete correction for total GAT error and its use without other corrections has questionable utility [9, 10]. The only true IOP is that measured by an invasive intracamerally placed pressure transducer. All other measurements are clinically feasible approximations of pressure with inherent biases from true intracameral IOP. The Goldmann applanation tonometry has been generally shown to significantly underestimate intracameral IOP both in vitro and in vivo [11–14]. Additionally, several studies have shown a direct correlation to corneal biomechanical parameters and the error produced by Goldmann applanation IOP measurement compared to true intracameral pressure [11–14]. Prior work has demonstrated a correlation in cadaver eye CCT as a measure of corneal hydration and shown an increase in corneal rigidity [8].

The present clinical study was designed to compare the Goldmann applanation tonometer to manometrically adjusted intracameral pressure to measure overall and positional error and sensitivity to corneal biomechanical parameters, both in vitro and in vivo*.*

## Methods

### Human eye surgical Intracameral IOP testing

A prospective intra-surgical clinical study was performed at Carondelet Foothills Ambulatory Surgery Center in Tucson, Arizona. Fifty eight (58) eyes (from 38 patients) aged 18 and older and were enrolled from the Arizona Eye Consultants clinic. A sample size of fifty eight (58) eyes was determined sufficient to demonstrate statistical correlation by one group single correlation with a probable correlation coefficient of *r* = 0.20 (alpha = 0.05). The prospective study enrolled patients scheduled for phacoemulsification, cataract surgery. A thorough ophthalmic exam was completed on all patients by one of two licensed investigators (SM, JL) to include slit-lamp biomicroscopy, anterior segment ocular coherence tomography (OCT) with central corneal thickness (CCT measurement (Zeiss HD-OCT, Jena, Germany), corneal topography (Zeiss Atlas model 9000 Jena, Germany), dilated funduscopy and an Ocular Response Analyzer (ORA) with corneal resistance factor (CRF) derived from corneal hysteresis (CH) measurements (Reichert Ophthalmic Instruments, Depew, New York). The study enrollment criteria included: (1) clinical indications for phacoemulsification (2) adequate patient target fixation (3) corneal curvature between 38.00 and 50.00 diopters (D); and (4) Less than 3.50 D of corneal astigmatism. Subjects were selected in accordance with the following exclusion criteria: Ocular surgery within the last 3 months; pregnant or nursing: only one functional eye; poor or eccentric fixation; high corneal astigmatism (>3.5 diopters); corneal scarring; microphthalmos; buphthalmos; severe dry eyes; blepharospasm; nystagmus; keratoconus; or any other corneal or conjunctival pathology or infection.

The research protocol conformed to the tenets of the Helsinki Declaration and was approved by Chesapeake Independent Review Board (IRB). All patients received a complete informed consent detailing risks of the study verbally and in writing.

Measurements were performed in the following order: CCT, topography, ORA, Applanation IOP with intracameral IOP. Each investigator was masked to the results of the other tests. Anterior segment OCT with CCT, corneal topography, and ORA with CRF were measured by a non-surgical investigator 1 day before surgery. With a spectral domain optical coherence tomographer HD-OCT, the corneal thickness at 3 locations was measured and averaged for analysis.

Corneal biomechanical properties were approximated by measurements with an ORA by a non-surgical investigator 1 day before surgery. Topical anesthetic drops were applied so that examination conditions were equivalent to other measurements in this study. CRF was measured in the siting position as an indicator of corneal biomechanical properties. CH results from the dynamic nature of the air pulse and the viscous damping inherent in the cornea. It was measured as the difference between the inward (P1) and the outward (P2) applanation pressures. CRF is an empirically derived measurement from CH of both the viscous and elastic resistance encountered by the air jet while deforming the corneal surface. It is equal to (P1 − 0.7P2) [6, 8]. ORA measurements were taken in triplicate, and the average value was taken for statistical analysis. Off-scale values were discarded, as well as measurements that could not be repeated three times. A Zeiss HD-OCT-5000 spectral domain optical coherence tomographer was used by the assistant to measure central corneal thickness. Finally, the assistant investigator completed a corneal topography and an averaged corneal curvature was used for analysis over the central 3 mm diameter of the cornea in accordance with ANSI Z80.23. The surgical investigator conducting IOP measurements was masked to the results of the assistant investigator’s tests.

A standard surgical prep and drape was completed followed by the initial surgical ocular incisions. Intracameral preservative-free lidocaine 1% (1cm^3^) was instilled in the anterior chamber. At this point, the disposable anterior chamber cannula (Sterimedix, Reddich, UK) was placed through the surgical paracentesis and checked to insure no leaks were present around the cannula. The Incision was 1.2 mm at a ‘near clear’ corneal location almost tangential to the limbus. The cannula and tubing were adjusted and secured throughout the measurements to eliminate any visible endothelial folds minimizing potential changes to the biomechanical properties of the central cornea.Surgical Balanced Salt Solution (BSS) was used to maintain and adjust the anterior chamber pressure by elevating bottle height (Alcon, Ft. Worth, TX). The intracameral surgical tubing was attached to a disposable right heart catheter pressure tranducer (Transpac IV, ICUMedical, San Clemente, CA)(accuracy +/−1%) and zeroed though the monitor (DatexOmeda S/5, Ge Healthcare, Chicago, Il) at a bottle height level with the anterior chamber of the surgical eye. Pressure Data was recorded at 25 Hz on S/5 Collect software (Ge Healthcare, Chicago, Il). Intracameral IOP was adjusted and allowed to stabilize at 10 mmHg as measured by the pressure transducer. Tear film was standardized by using Weck-cell sponge drying of the ocular fornices prior to measurement. A sterilized and daily calibrated Perkins (Goldmann type) tonometer (Perkins Tonometer MK2, Haag Streit, USA) was then used by the surgical investigator to measure applanation IOP at two averaged measurements each with the Perkins tonometer. Fluorescein (Fluorescein Sodium Ophthalmic Solution 0.25%/0.4%, Bausch & Lomb, Tampa, FL) was applied prior to each measurement so that examination conditions were equivalent. Measurements of IOP were made two (2) times with the Perkins tonometer (one measurement was considered by averaging measurements at 180 and 90 degrees to correct for astigmatism). If the sequential measurements with one prism were more than 2 mmHg different, then a third measurement was obtained. All three measurements were then averaged. The third measurements were included in the study if it was within the range of the first two, otherwise all measurements were discarded. The intracameral IOP was then adjusted and allowed to stabilize at 20 mm and 40 mmHg as measured by the pressure transducer and the IOP measurement was repeated with the Perkins tonometer. Eight (8) patients were randomly selected to measure IOP in both the supine and upright seated positions with the intracameral pressure set to 20 mmHg in both positions. This was completed to confirm the effects and correction for patient position on applanation tonometry which are demonstrated in the cadaver eye portion of the study described below.

Statistical analysis included pressure comparisons between the GAT and true intracameral pressure noting the average and standard deviation with Homeoscadastic two-tailed Student’s-t test to examine probable significance of the differences. Linear correlation coefficients were examined with the GAT IOP measurements versus measured error parameters of CCT and CRF. A multiple regression analysis was calculated to examine the effect of two independent error parameter variables; CCT and CRF (Dof = 3, 95%CI).

### Human cadaveric eye testing

Twenty one (21) enucleated human globes were obtained from the Georgia Eye Bank (Atlanta, GA). The whole globes were shipped less than 24 h post-mortem and stored at 4 °C in Optisol chambers until use [15]. All corneas were of corneal transplant quality without prior surgery. The cadaver eyes are used on the day of arrival within 36 h post mortem. The eyes, ages of the cadavers, and cause of death were recorded. Eyes with a history or evidence of previous anterior segment intraocular surgery (except cataract) or corneal abnormalities were excluded.

**Fig. 1:**
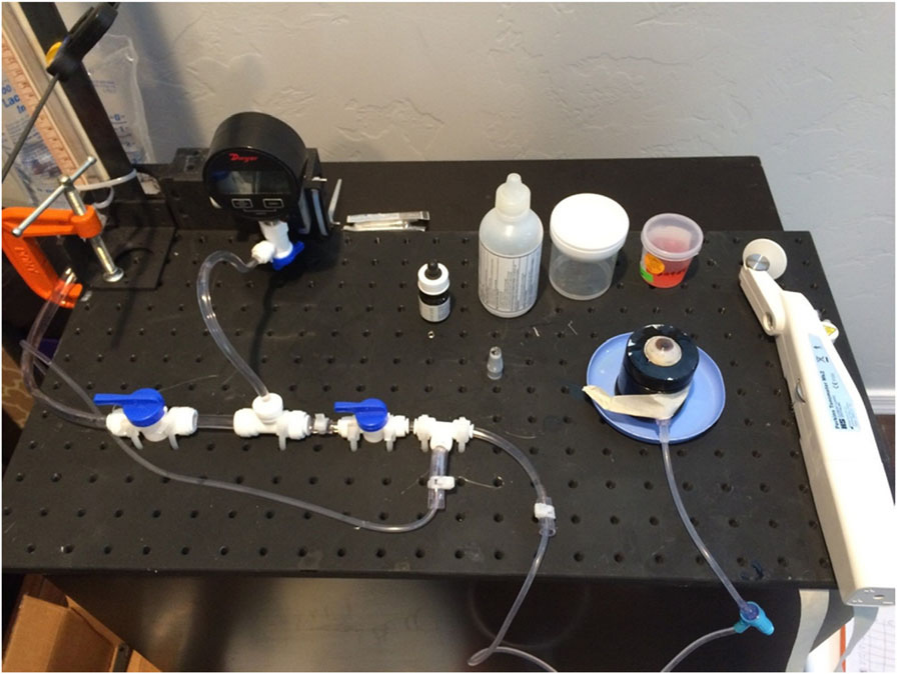
Ocular globe IOP apparatus for measuring IOP in the supine position, showing a Perkins type tonometer

They were stabilized in a specially designed apparatus for manometrically pressurizing and measuring IOP in a whole globe (Fig. 1) with the cornea exposed.

Standard biological precautions were followed when handling eye tissue. The corneal thickness was measured via Reichert pachymeter for IOP correlation to corneal thickness errors. The corneal thickness at central location was measured 3 times and averaged for analysis.

**Fig. 2:**
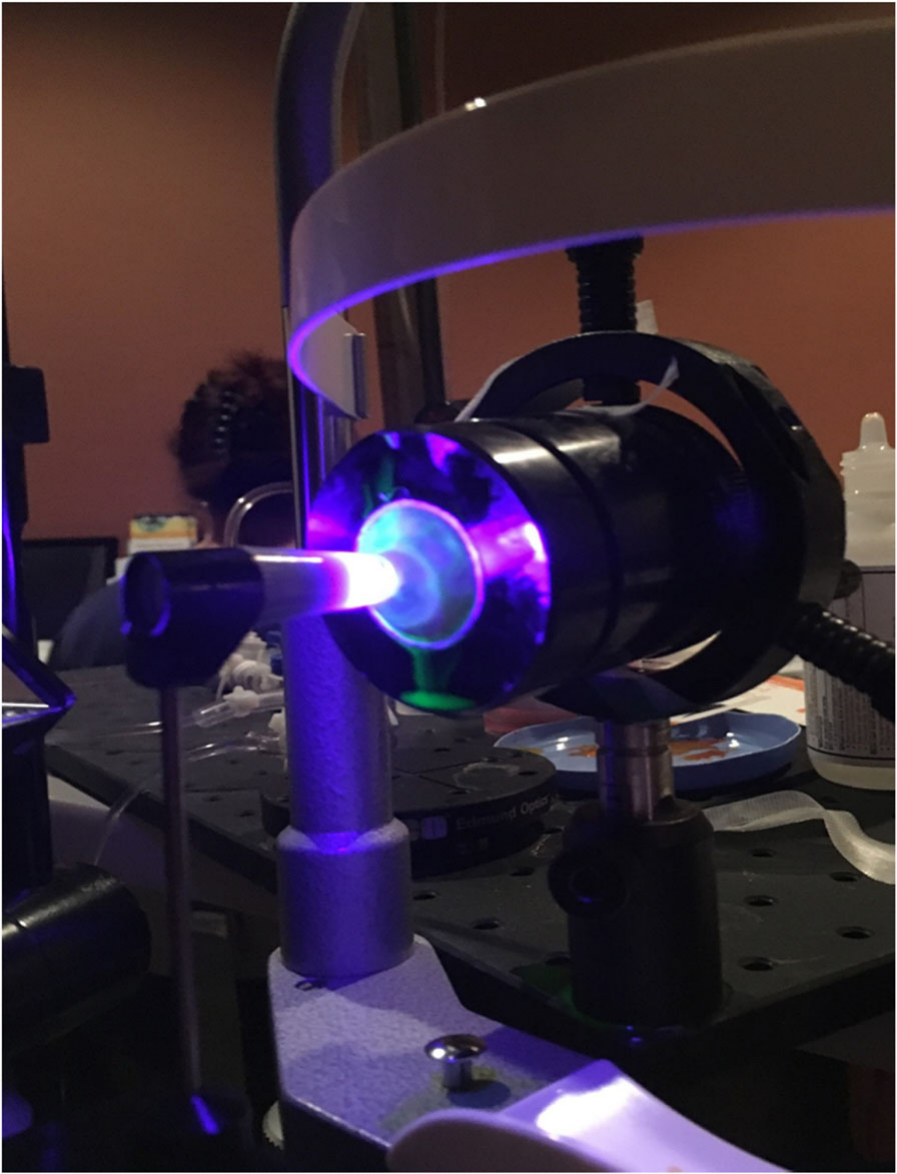
Ocular globe IOP apparatus for measuring upright IOP showing Goldmann type tonometer

All 21 eyes remained epithelized and hydrated with standard isotonic BSS. BSS was used to hydrate the corneal epithelium between measurements before the application of fluorescein solution. A 22-gauge needle with Y-adaptor (Saf-T-Intima, Vialon; Becton, Dickinson and Company, Franklin Lakes, NJ) was then inserted into the anterior chamber via a separate scleral approach. Extreme care was taken with all penetrations of the eye to avoid touching the endothelium, the iris, or the lens. The entire globe was mounted in the eye stabilization device shown in Fig. 1 embedded in moisturized gauze facing upward (supine) to be measured by the Perkins Tonometer MK2 (Haag Streit USA,). Subsequently, the IOP was measured at the same manometric pressure in the upright position with the Slit-lamp mounted Goldmann tonometer 900 (Fig. 2). The globe elevation at the central cornea was maintained equal in both supine Perkins and upright Goldmann measurement positions to insure a constant intracameral IOP. IOP measurements were completed only at a single intracameral pressure for each globe. The clinical equivalence in IOP measurement of the Perkins tonometer with the slit lamp mounted GAT has been established [16]. The needle IV tube was connected to a manometric transducer (Dwyer Instruments, Michigan City, IN), an isotonic sodium chloride solution infusion bottle, and an open-air reference tube.

Multiple stopcocks were attached to bleed all bubbles from the system and to allow either open or closed stopcock techniques (open used in study). The transducer and the anterior chamber were maintained at the same height for both upright and supine measurements. The isotonic sodium chloride solution infusion bottle was attached to a manually driven intravenous pole for bottle height adjustment.

IOP measurements were taken utilizing the Perkins tonometer for supine measurements and a slit lamp mounted GAT for upright measurements. Previous studies demonstrated that the Perkins Tonometer is clinically equivalent to the slit lamp mounted GAT [16]. Three (3) eyes were individually measured five (5) times by two (2) different examiners (10 total) with each prism at each of the following seven (7) intracameral pressures (5, 10, 20, 30, 40, 50, 60 mmHg). Each measurement consisted of a standard reference axis measurement averaged with a measurement rotated counter-clockwise 90 degrees from the standard reference axis to account for any astigmatic errors. For example, three cadaver eyes were measured 10 times at a 5 mmHg intracameral pressure in both the upright and supine positions (total of 20 measurements on each eye). A randomization occurred to determine which position was utilized first. BSS was used in the application of fluorescein solution to limit epithelial toxicity. After each series of measurements on an eye at a given pressure, the bottle height was lowered to the initial 4.8 cm. The series was only accepted if the initial and closing manometric pressures were within ±1 mmHg.

Statistical analysis included pressure comparison between the GAT prism and the true intracameral pressure noting the average and variance. Homeoscadastic two-tailed Student’s-t test was used to examine probable significance of the differences in IOP measurement between the supine and upright positions. Linear correlation coefficients were examined with the GAT IOP measurements versus measured error parameters of CCT and CRF. A multivariate regression analysis with a linear mixed-effects model was carried out to compare live human eye sensitivities of the GAT IOP reading errors to CCT and CRF. Cadaver eye CCT Correlation was examined as a possible correlation with post-mortem corneal hydration, but any other correlations were not possible on the post-mortem tissue and even post-mortem CCT may have little relation to live human cornea CCT.

## Results

Intraocular pressure measurements using the Perkins applanation tonometer on patients undergoing cataract surgery were completed on 58 eyes of 48 patients.

**Fig. 3:**
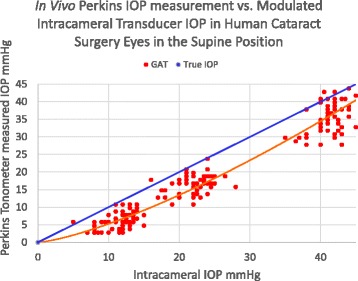
In Vivo Perkins IOP measurement Scatterplot over all Intracameral IOPs In live human eyes undergoing cataract surgery

A general linear mixed effects (GLME) analysis was completed and a post-hoc power calculation examined the complete independence verses complete dependence between those patients in which measurements were completed on bilateral eyes. The Power dropped from 99% to 91% when considering the bilateral measurements to be completely dependent. Complete dependence between bilateral IOP measurements is far from the case, therefore the power is likely somewhere between the extremes listed, both of which are adequate. The study’s average subject age was 66+/−8 years with 31 females and 27 males. The Perkins applanation IOP measured in the supine position was significantly less than the Intracameral transducer measured pressure at all three modulated pressures (10, 20, and 40 mmHg). See Fig. 3 illustrating the measured applanation IOP line under the true intracameral IOP line.

**Fig. 4:**
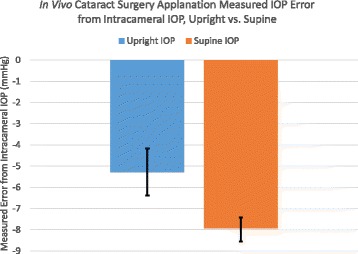
In Vivo Perkins IOP measurement error from true intracameral IOP in cataract surgery patients in both the upright and supine positions (95% CI)

The Perkins applanation tonometer measured (supine position), significantly less than the true intracameral IOP by −7.9 +/−2.3 mmHg (*p* = 0.001), Fig. 4. The measured IOP in the upright position also measured significantly less than the true intracameral IOP by −5.2 +/−1.6 mmHg (*p* = 0.03). In live surgical eyes with a controlled intracameral pressure, IOP measurement error is significantly less measuring in the upright position compared to the supine position by an average of 2.7 +/−1.3 mmHg (*p* = 0.04).

**Fig. 5:**
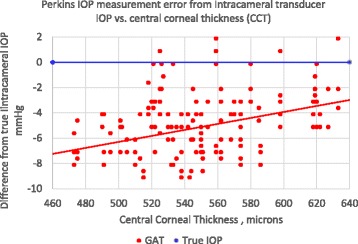
In Vivo Perkins IOP measurement error from true intracameral IOP correlated to central corneal thickness (CCT) in patients undergoing cataract surgery (corrected for supine to upright position error)

The Perkins tonometer IOP was measured in the surgical patients and correlated to CCT. The subject’s average CCT was 546+/− 40 μm which is comparable to a similar study at 556+/−40 μm [9]. Fig. 5 illustrates both the IOP measurement bias (corrected for upright position) and the slope sensitivity to CCT. The multivariate regression analysis with linear mixed-effects revealed a statistically significant (*p* = 0.029) sensitivity to CCT with the GAT at 0.024 mmHg/μmCCT.

**Fig. 6:**
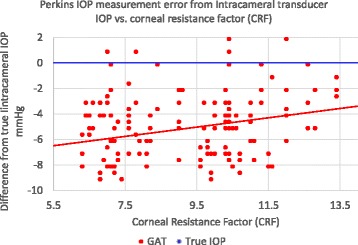
In Vivo Perkins IOP measurement error from true intracameral IOP correlated to corneal resistance factor (CRF) in patients undergoing cataract surgery (corrected for supine to upright position error)

The Perkins tonometer IOP was measured in the surgical patients and correlated to corneal resistance factor (CRF). The subject’s average CRF was 9.2 +/−2.1. Fig. 6 illustrates both the IOP measurement bias and the slope sensitivity to CRF. It demonstrates a linear error sensitivity of 0.37 mmHg/CRFunit with the GAT which is nearly statistically significant in the in the linear mixed effects analysis (*p* = 0.062).

Twenty one (21) human cadaver eyes were measured and analyzed each at a singular intracameral pressure. The average age of the donor was 59+/−19 years with 17 males and 4 females. All globes were intact with good corneal epithelium and without defect measured within 36 h post-mortem.

**Fig. 7:**
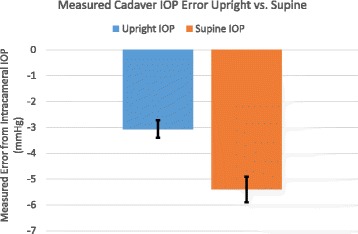
In Vitro IOP measurement error from true intracameral IOP in human cadaver eyes in both the upright (Goldmann measurement) and supine (Perkins measurement) positions

The Perkins applanation tonometer measured (supine position), significantly less than the true intracameral IOP by −5.4 +/−3.1 mmHg (*p* = 0.006), Fig. 7. The Goldmann measured IOP in the upright position also measured significantly less than the true intracameral IOP by −3.1+/−2.5 mmHg (*p* = 0.02). In fresh human cadaver eyes with a controlled intracameral pressure, IOP measurement error is significantly less measuring in the upright position compared to the supine position by an average of 2.3 +/−1.9 mmHg (*p* = 0.01).

**Fig. 8:**
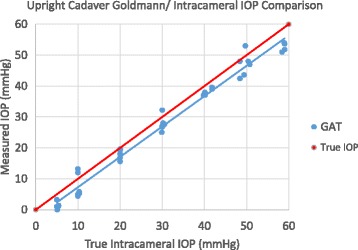
In Vitro Goldmann IOP measurement Scatterplot over all Intracameral IOPs In human cadaver eyes, upright position

The Goldmann applanation IOP measured in the upright position was significantly less than the Intracameral transducer measured pressure at all seven (7) modulated pressures (5, 10, 20, 30, 40, 50, and 60 mmHg). See Fig. 8 illustrating the bias in measured Goldmann applantion IOP line compared to the true intracameral IOP line.

**Fig. 9:**
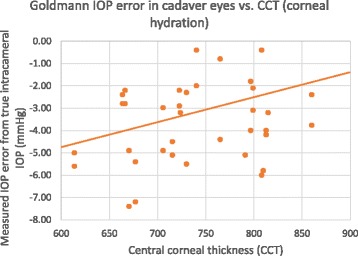
In Vitro Goldmann IOP measurement error from true intracameral IOP correlated to central corneal thickness (CCT) as a measure of corneal hydration in human cadaver eyes

The Goldmann tonometer IOP was measured in the cadaver eyes and correlated to CCT as a measure of corneal hydration Post-mortem. The donor’s average CCT was 748+/− 65 μm. Fig. 9 illustrates both the IOP measurement bias (Goldmann upright position) and the slope sensitivity to CCT. The linear correlation coefficient of the plot is low at *r* = 0.10 (*p* = 0.40), but appears to weakly demonstrate a linear error sensitivity of 0.012 mmHg/μCCT. Table 1 summarizes the findings.

**Table 1:** Summary of findings

Summary table	In vivo (live)	+/−SD	In vitro (Cadaver)	+/−SD
Upright IOP Error (mmHg)	-5.2	1.6	−3.1	2.5
Supine IOP Error (mmHg)	−7.9	2.3	−5.4	3.1
Difference Upright-Supine (mmHg)	−2.7	1.3	−2.3	1.9
CCT Sensitivity (mmHg/μmCCT)	0.024	0.18	0.012	0.1
CRF Sensitivity (mmHg/CRF unit)	0.037	0.08	–	–

## Discussion

Goldmann applanation tonometry and Perkins tonometry significantly underestimate true intracameral IOP. The lower applanation IOP measurements are also corroborated by other studies [10–14, 17]. One study has shown an over estimation in IOP measurements [18]. In one additional study, all averaged IOP measurements were almost exactly (<1 mmHg error) the same as intracameral pressure [19]. A critical difference in the design of this study is that the intracameral pressure was set (modulated) in vivo and adjusted to simultaneously compare to the applanation IOP measurement as opposed to a cannula placed to check the intracameral pressure and a subsequent IOP measurement taken. Moreover, the IOP measurement errors presented here were corroborated in two separate studies using an entirely different apparatus for in vivo and in vitro studies. A more difficult question to answer is: Why are the IOP measurements lower than intracameral IOP? According to thin-wall pressure vessel theory upon which the Imbert-Fick principle and Goldmann design is based, the IOP measurement should be exactly equal to the intracameral pressure for an infinitely thin (membrane) cornea and any added corneal rigidity force would then over-estimate the IOP measurement. In our prior mathematical modeling we attributed about -3 mmHg to the adhesion force of the tear film [17]. Our findings in the mathematical modeling showed an additional −3.5 mmHg error underestimating the intracameral IOP [17]. The additional biomechanical underestimation was due a buckling effect or dimpling in the central cornea when applanated flat, which contributed close to zero force to the tonometer prism face.

The in vivo and in vitro results indicate a correlation in IOP measurement error to CCT which corroborates prior intracameral IOP studies verifying the published Dresdner CCT correction [19]. The design of a new shaped tonometer prism face has been shown to significantly negate this CCT sensitivity [17].

The difference between the upright and supine measurements in an eye with a controlled constant intracameral pressure was demonstrated In vivo and in vitro. The supine position measurement adds significantly more error to the applanation IOP compared to the upright position. Several studies have examined the *clinical* differences in IOP measurement between upright and supine positions and have shown either the same or increased IOP when supine [20–22]. The critical difference in these studies and ours is found in the modulated control and of the intracameral pressure. The intracameral pressure will be at our desired set point in both upright and supine positions. This modulation negates any positional compensation of intracameral pressure by increased venous pressure or orbital Valsalva pressure. The reason for the lower pressure (and greater error) in the supine position is very likely related to the weight of the cornea. The difference of about 2.8 mmHg would be easily attributable to the added 280 mg of downward force (the equivalent weight of a small corneal transplant button) which would only effect the supine measurements.

Results of fresh human cadaver eyes compare well to intra-surgical live human eyes. The overall bias error is similar but somewhat less than in vivo IOP surgical measurements. Also, the difference between supine and surgical measurement is very close between cadaver and in vivo IOP measurements. The clinical equivalence in IOP measurement between the Perkins and Goldmann tonometers has been shown to be negligible and the authors did not see differences in the upright cadaver eyes measured by both tonometers [16]. Still there is a possibility of the cadaver eye upright-Goldmann measurement verses supine-Perkins measurement producing a confounding factor. The increased central corneal thickness average of 200 μm seen less than 36 h is indicative immediate endothelial function changes, post-mortem. It is likely that other post-mortem changes affecting the biomechanical behavior of the cornea account for the difference in IOP measurement error from intracameral pressure compared to live human eyes (−3.1 mmHg vs. −5.2 mmHg). The error response sensitivity to CCT is also similar between cadaver and in vivo eyes. Even though post-mortem CCT is related mainly to corneal hydration from reduced endothelial function, it likely has a basis in pre-mortem CCT affecting corneal biomechanical response [6, 8]. Low correlation coefficients are common in clinical IOP studies due to the multiple variables in measurement error such as corneal thickness, rigidity, curvature, patient age and tear film adhesion [6, 11, 13, 14]. The in vivo results were completed on a relatively older population averaging 66+/−8 years in which corneas have been shown to be relatively more rigid possibly skewing the results [6]. However, the corroborating results found in the somewhat younger in vitro population (57+/−19 years) indicate that the IOP measurement errors are consistent. There may be differences in substantially younger populations in which the IOP measurement errors have been shown to be significantly more pronounced [23]. The similar responses seen with fresh cadaver eyes allow for their use as a safe and less expensive substitute for live human eyes in many investigative studies examining IOP measurement.

The errors in Goldmann applanation type tonometry do not appear to be intuitive. There is a complex biomechanical response by the cornea and likely globe when the corneal surface is applanated. It is possible, with a careful understanding of these errors, to improve applanation tonometry accuracy in both overall bias and errors due to patient variability such as CCT. Additionally, this improved accuracy may translate into added benefits to both pediatric populations and veterinary applications in which these errors appear magnified [13, 15, 23].

## Abbreviations

BSS: Balanced salt solution
CCT: Central corneal thickness
CH: Corneal hysteresis
CRF: Corneal resistance factor
GAT: Goldmann applanation tonometer
IOP: Intraocular pressure
OCT: Optical coherence tomography
ORA: Ocular response analyzer
